# International Consortium to Advance Cross-Species Extrapolation of the Effects of Chemicals in Regulatory Toxicology

**DOI:** 10.1002/etc.5214

**Published:** 2021-10-25

**Authors:** Carlie A. LaLone, Niladri Basu, Patience Browne, Stephen W. Edwards, Michelle Embry, Fiona Sewell, Geoff Hodges

**Affiliations:** a Great Lakes Toxicology and Ecology Division, Center for Computational Toxicology and Exposure, Office of Research and Development, US Environmental Protection Agency, Duluth, Minnesota, USA; b Faculty of Agricultural and Environmental Sciences, McGill University, Montreal, Quebec, Canada; c Environment Directorate, Organisation for Economic Co-operation and Development, Paris, France; d GenOmics, Bioinformatics, and Translational Research Center, RTI International, Research Triangle Park, North Carolina, USA; e Health and Environmental Sciences Institute, Washington, District of Columbia, USA; f National Centre for the Replacement, Refinement, and Reduction of Animals in Research, London, UK; g Safety and Environmental Assurance Centre, Unilever, Sharnbrook, Bedfordshire, UK

## INTRODUCTION

Regulatory decisions surrounding chemical safety are based on human and environmental (ecological) protection goals. Historically, such decisions have relied on data from animal toxicity testing to inform hazard and risk assessment and determine whether chemicals pose a threat to human or environmental health. Traditionally, mammalian toxicity test data have driven human health considerations, and studies from select species representing different taxa have driven ecological considerations. Crosstalk and collaboration between human and ecological health knowledge-streams have been limited. This represents a barrier for realizing the ultimate protection goal, which is the health of the planet and all its inhabitants, as exemplified by the One Health approach (https://www.cdc.gov/onehealth/). However, there are global efforts within governments, nongovernmental organizations, academic research organizations, and industry sectors to bridge this gap and focus on achieving optimal health outcomes without the need for animal testing through recognition of the interconnectedness between people and all species that share the environment. With this in mind, it is recognized that focused and concerted efforts to advance methods for cross-species extrapolation that leverage existing toxicity data from both mammals and other model organisms can be used to protect all species.

To expedite the development and regulatory acceptance of computational methods, particularly bioinformatics, for informing cross-species extrapolation for the evaluation of chemical safety, there is a need to bring together tool/database/method developers and regulators in a global cross-sector collaborative consortium. These collaborations will help define regulatory needs, spark the creation of a bioinformatics toolbox, demonstrate the utility of various tools through coordinated application, and enhance communications with various stakeholders. The International Consortium to Advance Cross-Species Extrapolation in Regulation (ICACSER; https://www.setac.org/page/scixspecies) is being developed to align with both the One Health approach and the shifting paradigm in regulatory toxicology articulated by the National Research Council in 2007. Specifically, a strategy was described to include more efficient and cost-effective toxicity testing that takes advantage of cell-based and computational approaches for evaluating chemical safety in the 21st century (National Research Council, 2007). Such methods move away from the whole-animal testing that historically focused on apical endpoints, such as reproduction, growth, development, and mortality, toward testing molecular-, cellular-, and organ-level changes that can be predictive of upstream apical changes in biology and used for regulatory decision-making (National Research Council, 2007). It was envisioned that such a shift in toxicology would simultaneously reduce animal use. The objective of the present Focus article was to describe the challenges surrounding cross-species extrapolation in regulation and introduce new approach methods (NAMs) in bioinformatics that can enhance and broaden the ability to extrapolate toxicity knowledge beyond model organisms to the diversity of species through efforts lead by the developing ICACSER (Textbox 1).

## THE CHANGING REGULATORY LANDSCAPE

The global regulatory landscape is currently experiencing an evolution in thinking surrounding animals in toxicity testing, with the aim of eliminating or greatly reducing their use in toxicology. It has been 60 years since Russel and Burch developed the 3Rs (Replacement, Reduction, and Refinement) principles, providing a framework for performing more humane animal research (Burden et al., 2015). These principles have since been incorporated into test guidelines and legislation around the world, primarily to ensure that when conducted, experiments use the fewest animals necessary to answer the question at hand and maintain animal welfare standards.

In recent years, attention has focused on replacement opportunities, because there is recognition that traditional animal models (e.g., rodents) are not always the best predictive system for humans, or as surrogates for other species of concern. For example, in 2013 a change to legislation in Europe banned the marketing of personal care products containing ingredients that have been tested on animals (https://ec.europa.eu/health/sites/default/files/endocrine_disruptors/docs/cosmetic_1223_2009_regulation_en.pdf), and legislation to ban animal testing for cosmetic safety has subsequently been enacted in many other countries worldwide (Burden et al., 2015). Recent legislation has underscored the willingness of authorities to use and consider in vitro and other NAMs for regulatory safety evaluation. For example, in 2017, the European Union Registration, Evaluation, Authorisation and Restriction of Chemicals (REACH) regulatory information requirements were amended to make animal testing the last resort for filling data gaps. In 2019, the US Environmental Protection Agency (USEPA) Administrator issued a directive to eliminate mammalian regulatory and research studies completely by 2035, with associated funds to develop alternative methods (https://www.epa.gov/research/administrator-memo-prioritizing-efforts-reduce-animal-testing-september-10-2019). Even more recently, the Scientific Committee on Consumer Safety (2021) produced its 11th revision of guidance for the testing of cosmetic ingredients to provide greater focus on the application of NAMs. Government pledges and changes to legislation such as these, along with a growing appreciation of the need to further develop relevant and predictive methods for safety assessment that do not necessarily rely on animals, show that the data landscape is also changing. There is now greater focus on the generation of mechanistic, cell-based, and computationally derived information for consideration as alternatives to animal testing.

This shift is exemplified by data collected from available ecotoxicology literature curated in the ECOTOXicology Knowledgebase (ECOTOX; https://cfpub.epa.gov/ecotox/) from the 1980s to the present. The number of growth and mortality studies added to ECOTOX have remained relatively consistent throughout the last four decades, whereas increases have been observed in the reporting of molecular and cellular effects data since 2000. To facilitate the use of the increasing influx of mechanistic data to establish causal links to apical changes in individuals or populations, the adverse outcome pathway (AOP) conceptual framework is being considered or actively adopted in many regulatory, industry, and academic settings (Ankley et al., 2010).

## A ROLE FOR THE AOP FRAMEWORK IN SPECIES EXTRAPOLATION

The AOP framework is an approach for gathering existing pathway-based knowledge and developing causal linkages between levels of biological organization allowing for prediction of adverse effects of regulatory significance (Ankley et al., 2010). The AOP framework was designed to help remove the silos between human health and ecological assessments by allowing mammalian data to provide insights into potential effects on nonmammalian species and vice versa. This is accomplished by defining the taxonomic domain of applicability with an emphasis on the structural and functional conservation (or lack thereof) of biological mechanisms across species for the purpose of understanding how broadly available knowledge can be extrapolated from one species to others. Adverse outcome pathways provide the opportunity to extrapolate effects across species, qualitatively and quantitatively, through the identification of conserved early events in the AOP, termed molecular initiating events (MIEs), in which the chemical interacts with a biomolecule, as well as at subsequent key events and key event relationships (KERs) as the pathways move from the molecular level to cells, organs, and tissues out to individuals and populations (Villeneuve et al., 2014). The demonstration of conservation across species at these various levels of biological organization may decrease the numbers and diversity of species needed for toxicity testing, including nonhuman primates. For example, if evidence exists that early pathway events are structurally and functionally conserved across vertebrates, then additional testing in more vertebrate species may not be necessary to gain further information to make the causal linkages across early key events in the AOP framework. Similarly, if there is strong evidence of, for example, chemical–protein interactions in vertebrate species combined with evidence of a lack of conservation in invertebrate species, this knowledge could reduce the need for additional toxicity testing in invertebrate species.

From this perspective, it is not only the target or surrogate species that is considered, but also the conservation of the biological pathway in the context of species for which that pathway is relevant. This shift in perspective allows more effective use of existing toxicological data and improved efficiency in chemical safety assessments by reducing the number of, and reliance on, toxicity tests in animals. However, there are still key challenges that need to be resolved to fully capitalize on the application of AOPs for extrapolating across species for risk assessment purposes. Perhaps the most notable of these challenges are the need to increase knowledge on the extent of functional conservation of downstream effects across species and the need to recognize that some adverse biological responses will be caused by multiple MIEs and/or multiple pathways comprising biological networks and/or by toxicokinetic considerations (Rivetti et al., 2020).

## CROSS-SPECIES EXTRAPOLATION IN CHEMICAL SAFETY ASSESSMENT

**FIGURE 1:**
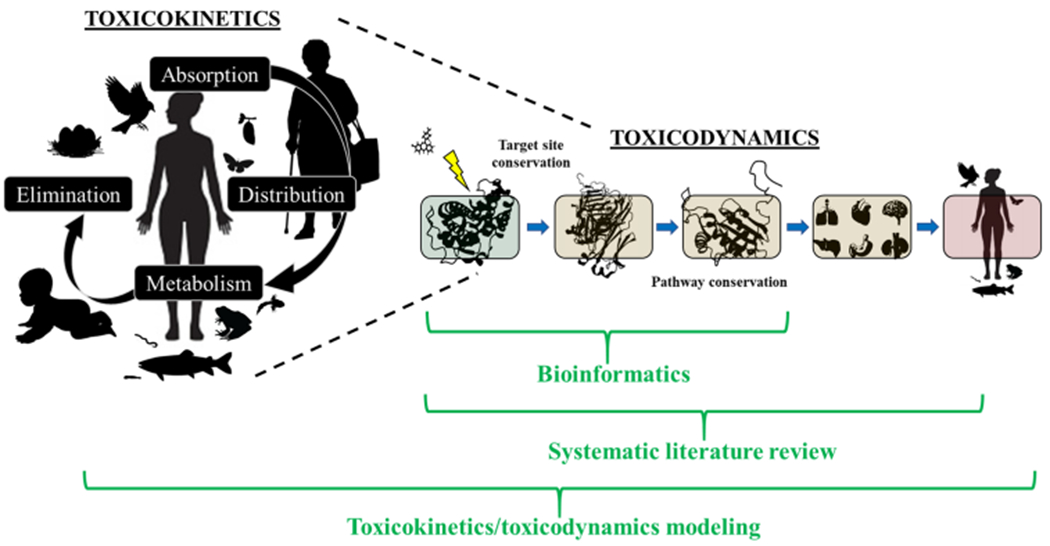
The recognized complexity for meaningful extrapolation of toxicity knowledge across species includes both toxicokinetic and toxicodynamic considerations.

Extrapolating toxicity from one species, typically a model organism, to others, considering both toxicokinetics and toxicodynamics, is extremely challenging (Figure 1). From an environmental risk assessment (ERA) standpoint, this complexity is exemplified by the overarching aim to protect ecosystems that are comprised of a diverse range of species, each with potentially different sensitivities to the array of chemicals and other stressors to which they may be exposed. Simplifying this complexity has led to the use of extrapolation factors and species sensitivity distributions (SSDs) in ERAs (Spurgeon et al., 2020). Factor-based extrapolation approaches are generalized and do not consider physiological, spatial, or temporal species differences, whereas SSDs are determined using cumulative distributions of measured species sensitivity (often expressed as toxicity values). Although SSDs have a long history of use in helping to determine safe limits of chemical exposure, they suffer from a necessary trade-off between the use of high-quality data and the need for toxicity information from a large number of species. Although both approaches are practical tools supporting chemical safety decision-making, they overlook the details of pathway-based similarities and differences that dictate taxon-specific sensitivity to stressors. Taxa-specific differences become especially important when a protection goal is necessary for a threatened or endangered species.

An understanding of chemical exposure, including absorption, distribution, metabolism, and elimination (ADME), and the organism’s life stage, life history, and traits is a major consideration relative to cross-species extrapolation of effects. A number of studies link differences in biological traits to the differences in species’ responses to chemical exposure (Spurgeon et al., 2020). Traits related to differences observed in ADME, including species behavior, surface area-to-body mass scaling relationships, and metabolic capacities, are particularly critical in this respect. Understanding how differences in ADME among species impact internal chemical concentrations at target sites is critical to fully implement mechanistically based cross-species extrapolation for risk assessment. If internal concentrations of a chemical fail to reach a threshold activation concentration at the target, a MIE will not be triggered and the AOP will not proceed to the adverse effect. Conversely, chemical concentrations substantially above a threshold may lead to general disruption of membranes and molecular processes and cell stress in what has been termed the cytotoxic burst (CTB) phenomenon with respect to in vitro assays, which can mask or overwhelm more specific pathway perturbations observed at lower concentrations (Judson et al., 2016). Similar observations have been made when considering narcotic effects pertaining to in vivo studies. Additional complexity comes from differences in toxicokinetics/metabolic rates across different cell types, tissues, individuals, and species. Other factors determining species sensitivities to chemicals include the life stages of organisms and whether there was an acute or chronic exposure. These become critical considerations to enable the replacement of traditional in vivo test systems with in vitro (e.g., cell-based) assays or other NAMs, such as omics, on both an intra- and interspecies level. Thus, from an applied risk assessment perspective, cross-species extrapolation cannot be divorced from an understanding of the specific exposure scenario, although screening level assessments may be more flexible.

Although the magnitude of the challenge to connect potential for exposure to potential for effect in risk assessment should not be underestimated, the number of available models to better characterize and understand chemical concentrations and distribution within organisms is growing. Tools such as the MERLIN-Expo software (https://merlin-expo.eu/) and Gastro-Plus (https://www.simulations-plus.com/software/gastroplus/) provide increasingly good mechanistic modeling approaches for simulating chemical distribution in humans. More recent physiologically based pharmacokinetic models in fish provide options for increased cross-species extrapolation (Rivetti et al., 2020). Nonetheless, there remains a significant research challenge to address toxicokinetic modeling for less well-characterized species.

In addition, cross-species extrapolation of chemical effects depends heavily on knowledge of taxonomic conservation of key biological pathways (Table 1). For the most part, these considerations are either qualitative or neglected in decision-making, primarily because empirical data are lacking for the majority of species and cannot readily be generated. However, more detailed considerations of exposure and effects across the diversity of life are becoming more attainable for crossspecies extrapolation due to advances in informatics, including bioinformatics, systematic methods, and toxicokinetic and toxicodynamic modeling.

## THE USE OF MODEL ORGANISMS IN REGULATORY DECISION-MAKING

Model organisms have served as surrogates that provide empirical data for regulatory actions including risk assessment and have been the cornerstone of historical toxicity testing. These data are also used to demonstrate the predictivity of NAMs, such as high-throughput technologies, omics, organs on a chip, in vitro and subcellular fraction assays, and AOPs. Toxicity testing with mammalian species such as nonhuman primates, rat, mouse, rabbit, guinea pig, dog, sheep, and pig have been used for human health risk assessment. Nonmammalian species such as fathead minnow, rainbow trout, Japanese medaka, zebrafish, Japanese quail, African clawed frog, cladocerans, and green algae have been used historically to represent diverse taxonomic groups and trophic levels in the context of ERA. These species often were selected as model organisms due to characteristics that make them amenable to laboratory testing (e.g., developmental windows, ease of maintenance, and generation time) rather than their appropriateness as surrogates to represent toxicity in other species. The sensitivity of an organism to a chemical stressor has been assumed to be a function of their relatedness, but evolutionary relationships have yet to be consistently considered in extrapolating from surrogate species in the context of chemical safety evaluations.

The NAMs provide innovative and enhanced opportunities to accelerate chemical safety assessment and ensure that a risk assessment is grounded in biology rather than a reliance on a small and exclusive set of regulatory animal tests. As the need for rapid chemical screening and testing with reduced reliance on animals increases, there are opportunities to exploit existing data at several levels of biological organization (e.g., from sequence data to transcriptomics analyses to historical whole-organism studies) to define the taxonomic domain of applicability for biological pathways. This information can be used in rapid, cost-effective computational approaches for regulatory decision-making.

The AOP framework facilitates cross-species extrapolation based on pathway conservation through the definition of key events at each level of biological organization. Because the taxonomic applicability of each KER is defined by conservation of neighboring upstream and downstream key events, existing toxicological data can be incorporated into a chemical assessment to complement results from NAMs that primarily focus on early key events. The development of quantitative AOPs that incorporate thresholds required for a pathway to progress from one key event to the next will help to increase our understanding and will be critical for development and integration of NAM data into regulatory decision-making. Integrated approaches to testing and assessments that combine existing toxicological data from structurally related chemicals for downstream key events with bioactivity results from NAMs can increase confidence in toxicity predictions for chemicals for which data are limited. In tiered testing scenarios, the presence or absence of existing data will help guide additional testing when needed, with a focus on those species most likely to be susceptible. A benefit with this approach is that results from assays conducted with new chemicals can be incorporated into the larger body of toxicological knowledge to increase our ability to make confident predictions based solely on NAMs.

## BIOINFORMATICS IN NAMs

The predictive computational tools that have been most frequently used by safety assessors and regulators for understanding the mode and mechanism of action of chemicals are predominantly centered around predictions of quantitative–structure activity relationship (QSAR) models based on chemical structure. More recent strategies have focused more on taxonomic relevance and mechanistic plausibility of predictions, therefore increasing opportunities for understanding mechanisms across species (Sapounidou et al., 2021). However, these QSAR approaches are still fundamentally seated in determining structural (i.e., chemical fragment) alerts. The application of QSARs can be enhanced by closer integration with approaches that exploit the wealth of biological and mechanistic knowledge now available. Fortunately, the shifting paradigm in toxicity testing has inspired the use of bioinformatics and specifically the development of tools and workflows for computationally predicting the taxonomic relevance of existing and newly generated toxicity data across species. For example, methods/tools such as the USEPA’s Sequence Alignment to Predict Across Species Susceptibility (SeqAPASS; https://seqapass.epa.gov/seqapass/) tool, ECOdrug (http://www.ecodrug.org/), Seq2Fun (https://www.seq2fun.ca/), and other phylogenetic comparative methods and trait-based workflows have been released to the public, peer-reviewed, and published (Spurgeon et al., 2020). However, there is recognition that although each of these tools/approaches brings important information to the challenge of cross-species extrapolation, no individual method in isolation is capable of advancing the science and use of these types of data in regulatory decision-making.

The utility of the data generated from NAMs is enhanced through integration with other information from laboratory-based experimentation and computational approaches to deliver a comprehensive picture, considering toxicokinetics and toxicodynamics for understanding species relatedness. There are opportunities to apply systematic methods for literature review using text mining and machine learning, which allow for more rapid and thorough searching, categorizing, and prioritizing of the literature. The resulting information can be used to evaluate conservation of biological structure and function and support pathway-based taxonomic domains of applicability. It is envisioned that a strategic combination of published, transparent, and scientifically sound methods for cross-species extrapolation, supported by empirical evidence, will enhance the utility of approaches to understand cross-species extrapolation for regulatory applications and acceptance in this modern era seeking alternatives to animal testing.

Workflows that use state-of-the-science bioinformatics approaches originating from drug discovery and development are beginning to be applied to chemical safety assessments. For example, homology modeling that computationally creates protein models from existing structures has been used to generate protein structures from diverse taxa that can be used in molecular docking and molecular dynamic simulations (Cheng et al., 2021). Molecular dynamic simulation provides an opportunity to calculate the free energy of binding, which can be compared with empirically derived binding affinity results. These advances in computational power to evaluate protein–-chemical and protein–protein interactions can be applied for species extrapolation purposes in the context of biological pathways. This provides greater resolution in understanding species similarities and differences in binding, which could add a quantitative value to predictions of chemical susceptibility across the diversity of species. As predictive toxicology using bioinformatics for cross-species extrapolation moves down the continuum from protein sequence to structure to function, examples demonstrating the utility of these methods can add value when compared with standardized test methods historically used for regulatory decision-making (Textbox 2).

The AOPs are well suited to align predictive approaches for species extrapolation to decision-making, in the context of serving as an organizing framework for these efforts. Existing tools, although they may not yet have been applied to the challenge of cross-species extrapolation, can be classified by the level of biological organization they cover (Table 1), from molecular-level events to population. The AOP framework can guide integration efforts for tools that address the same area of biological organization, can help to define data gaps when a given level is inadequately addressed, and can facilitate the integration of information across the different levels of biological organization. The development of bioinformatic tools to increase confidence in cross-species predictions will promote the use of existing data collected from many species when one is performing a chemical risk assessment, while simultaneously facilitating the risk assessments performed for a variety of species.

## ICACSER

To advance cross-species extrapolation and uphold regulatory goals for assessing human and ecological health without animal testing, a global, cross-sector consortium, the ICACSER, has been created including researchers, regulators, and other advocates working to integrate bioinformatics approaches. Although the challenges in species extrapolation will not be addressed solely by bioinformatics, as an initial focus, advancing the use of bioinformatics will lay the foundation for broader integration of methods that can fill knowledge gaps in species extrapolation across the exposure/effect continuum (e.g., toxicokinetic/toxicodynamic models). The specific near term goals of the ICACSER are fourfold:

1. Develop an inventory of available, peer-reviewed, state-of-the-science tools for defining the taxonomic domain of applicability and propose ways to integrate data streams that inform challenges in species extrapolation.
2. Define the global regulatory landscape and create a vision for exploiting cross-species extrapolation of toxicity knowledge to support risk-based chemical safety decision-making across both human health and the environment.
3. Create a publicly accessible bioinformatics toolbox that integrates data-streams for consistent cross-species extrapolation of toxicity knowledge in a human and environmental health context.
4. Promote the adoption of integrated bioinformatic approaches to cross-species extrapolation by academia/regulators/industry/nongovernment organizations (NGOs) to inform human health and ecological chemical safety assessment.

The overarching purpose of this consortium will be to meet the needs of regulatory decision-makers and facilitate methods for species extrapolation essential for the optimal use of existing toxicological data and increased confidence in NAMs for toxicity testing (Textbox 3).

The ICACSER is currently run through a steering committee overseeing a number of key activity areas. The Steering Committee is meant to have an inclusive membership covering all interested regulatory, academic, industrial, and NGO organizations and including both human health and environmental interests. Currently the Steering Committee is comprised of representatives from the USEPA, the OECD, McGill University, RTI International, the Health and Environmental Sciences Institute (HESI), Unilever, and the National Centre for the Replacement, Refinement, and Reduction of Animals in Research (NC3Rs) and holds meetings approximately bimonthly to coordinate activities. Current activities are centered around the regulatory community to identify needs and opportunities for application of cross-species approaches. In parallel, the Committee has begun to identify and collate available tools and the coordination of a toolbox development using common architecture for integration of tools. Communication is a key element of the ICACSER outputs through both scientific media and applied regulatory mechanisms (e.g., updates of relevant guidance, etc.).

## CONCLUSIONS

Better use of existing animal model data to inform chemical safety assessments for regulatory decision-making is a necessity as toxicology evolves toward less animal testing. To advance beyond current practices in cross-species extrapolation and take advantage of existing knowledge that can be applied to a larger number of species, it is timely to bring together a global consortium with dedicated experts in the field including both researchers and decision-makers, to focus efforts. While recognizing the importance of toxicokinetics in understanding and applying cross-species extrapolation approaches, the initial focus of the ICACSER is on capitalizing on the rapidly expanding opportunities in bioinformatics methods for advancing regulatory decision-making. If successful, this will allow risk assessors to make better use of existing toxicological information and more easily consider the impact of chemicals on a variety of species.

## Figures and Tables

**TABLE 1:** Bioinformatics approaches that could be applied to challenges in cross-species extrapolation, aligning with biological levels of organization in the adverse outcome pathway framework

Level of biological organization	Approaches	Examples
Molecular	Sequence conservation of molecular targets Conservation of biological pathways	SeqAPASS (https://seqapass.epa.gov/seqapass/) ECOdrug (http://www.ecodrug.org/) Seq2Fun (https://www.seq2fun.ca/)
Cellular	Conservation of cell types within conserved tissues/organs Conservation of different biological pathways within a cell type	MetaboAnalyst (https://www.metaboanalyst.ca/) BMDExpress (https://www.sciome.com/bmdexpress/) FastBMD (https://www.fastbmd.ca/) NetworkAnalyst (https://www.networkanalyst.ca/) EcoToxXplorer (https://www.ecotoxxplorer.ca/) OmicsNet (https://www.omicsnet.ca/)
Tissue/organ	Conservation of tissues and organs Phenotypic relationships among species	The Monarch Initiative (https://monarchinitiative.org)
Organism	Phylogenetic relationships among species	T-Rex (http://www.trex.uqam.ca/) MEGAX (https://www.megasoftware.net/) PhyML (http://atgc.lirmm.fr/phyml/) RAxML (https://github.com/stamatak/standard-RAxML) NGPhylogeny (https://ngphylogeny.fr/) IQTree (http://www.iqtree.org/)
Population	Population-sequencing approaches Metabarcoding	VSEARCH (https://github.com/torognes/vsearch) SWARM (https://github.com/torognes/swarm)

## Data Availability

Data, associated metadata, and calculation tools are available from the corresponding author (LaLone.carlie@epa.gov).

## TEXTBOX 1: Coming to terms

**Table:**

Term	Definition
Adverse outcome pathway	Conceptual construct that portrays existing knowledge concerning the linkage between a direct molecular initiating event (e.g., a molecular interaction between a xenobiotic and a specific biomolecule) and an adverse outcome at a biological level of organization relevant to risk assessment.
Bioinformatics	The collection, organization, storage, analysis, and synthesis of biological information using computers commonly applied to -omics data such as molecular genetics, genomics, and proteomics.
Cytotoxic burst	Stress responses are activated in a nonspecific way at concentrations close to cell death.
One Health	Collaborative effort of multiple disciplines—working locally, nationally, and globally—to attain optimal health for people, animals, and the environment.
Species extrapolation	The act of using existing knowledge about one species to estimate, predict, project, or infer the effect, impact, or trajectory of another species.
Species sensitivity distribution	A cumulative probability distribution of a chemical’s toxicity measurements obtained from single-species bioassays of various species that can be used to estimate the ecotoxicological impacts of a chemical.
Systematic literature review	A predefined, systematic method whereby literature is assembled, reviewed, and assessed using standardized techniques.
Taxonomic domain of applicability	In the context of the adverse outcome pathway framework, it defines how broadly across taxa/species/subspecies the pathway knowledge is applicable based on conservation of structure and function.
Toxicokinetics	The rate of uptake, excretion, and metabolism of a chemical in an organism.
Toxicodynamics	The dynamic interaction of a chemical with a biological target resulting in a biological effect.
New approach methodologies (NAMs)	An umbrella term used to capture approaches or assays that reduce animal use and improve toxicokinetic or toxicodynamic knowledge of chemicals. They include in silico, in chemico, in vitro assays, omics, and toxicokinetic and exposure prediction.
Metabarcoding	The barcoding of DNA/RNA for the simultaneous identification of many taxa within the same sample.
Organisation for Economic Co-operation and Development (OECD)	An intergovernmental economic organisation with 37 member countries that works cooperatively to establish evidence-based international standards and find solutions to an array of social, economic, and environmental challenges.
Registration, Evaluation, Authorisation and Restriction of Chemicals (REACH)	A regulation of the European Union that was adopted to improve the protection of human health and the environment from the risks that can be posed by chemicals.

## TEXTBOX 2: Case examples demonstrate the use of bioinformatics for decision-making

The current practice in developing tools and workflows for using bioinformatics for decision-making is to describe the methods and functionality and apply the work to specific decision-making scenarios. For example, applications of the SeqAPASS tool have been published and applied to challenges in species extrapolation ranging from understanding pollinator and beneficial insect susceptibility to various pesticides, to extrapolating high-throughput screening results to other organisms in the context of the Endocrine Disruptor Screening Program in the United States, and more recently, to evaluating bioaccumulation potential for per- and poly-fluoroalkyl substances (https://www.epa.gov/chemical-research/sequence-alignment-predict-across-species-susceptibility). By working with risk assessors and regulatory decision-makers throughout the process of development of the tool, providing training opportunities, and demonstrating case examples relevant to current decision-making needs with associated peer-reviewed publications, SeqAPASS has received recognition as a tool that is ready for use in a nonanimal testing regulatory environment. In 2018, the OECD published the *Revised Guidance Document 150 on Standardised Test Guidelines for Evaluating Chemicals for Endocrine Disruption*, which recommended the SeqAPASS tool for evaluation of protein conservation for cross-species extrapolation (https://www.oecd.org/publications/guidance-document-on-standardised-test-guidelines-for-evaluating-chemicals-for-endocrine-disruption-2nd-edition-9789264304741-en.htm). In addition, the methods for evaluating protein conservation that are integrated into SeqAPASS were listed in the appendices of the White House National Strategy to Promote the Health of Honey Bees and Other Pollinators in 2016 as one of the efforts in Pollinator Research for the US Federal Government.
